# Identification of DBCCR1 as a suppressor in the development of lung cancer that is associated with increased DNA methyltransferase 1

**DOI:** 10.18632/oncotarget.15826

**Published:** 2017-03-02

**Authors:** Guoren Zhou, Jinjun Ye, Ying Fang, Zhi Zhang, Jingyuan Zhang, Lei Sun, Jifeng Feng

**Affiliations:** ^1^ Department of Chemotherapy, Jiangsu Cancer Hospital Affiliated to Nanjing Medical University, Nanjing 210000, Jiangsu, China; ^2^ Department of Radiotherapy, Jiangsu Cancer Hospital Affiliated to Nanjing Medical University, Nanjing 210000, Jiangsu, China; ^3^ Department of Thoracic Surgery, Jiangsu Cancer Hospital Affiliated to Nanjing Medical University, Nanjing 210000, Jiangsu, China; ^4^ Department of Pathology, Jiangsu Cancer Hospital Affiliated to Nanjing Medical University, Nanjing 210000, Jiangsu, China; ^5^ Department of Medical Iconography, Jiangsu Cancer Hospital Affiliated to Nanjing Medical University, Nanjing 210000, Jiangsu, China

**Keywords:** lung cancer, DNA methyltransferase, tumor suppressor, DBCCR1, epigenetics

## Abstract

Accumulating evidence has pointed to a role of the CpG island hypermethylation in the regulation of cancer-related genes in tumor progression. However, the biological impacts in cancer pathogenesis associated with down-regulation of such gene targets remains elusive. Here we focused on a potential target of hypermethylation, DBCCR1 (deleted in bladder cancer chromosome region 1), a gene encoding a candidate tumor suppressor. We found that the expression of DBCCR1 is significantly lower in the lung cancer tissues compared with adjacent non-tumor tissues of patients. Importantly, the decreased DBCCR1 was found correlated with more advanced stages of cancer, and with a significantly shorter survival of patients. Genetic silencing DBCCR1 in human lung cancer cell line A549 resulted in an enhanced proliferation, migration, and invasion capacity. Conversely, restoring DBCCR1 expression blocked the growth and inhibited the ability of cancer cell in migration and invasion. Interestingly, DBCCR1 attenuates the expression of DNMT1 (DNA methyltransferase 1), suggesting a reciprocal regulation between genetic silencing of cancer suppressor genes and activating DNA methylation. Our data thus implicates DBCCR1 downregulation as a potential module in the pathogenesis of lung cancer through DNA methylation.

## INTRODUCTION

Genetic abnormalities are key integral components of cancer pathogenesis, and numerous genetic alternations have now been found associated with human cancers [1]. Among them, epigenetic changes have been proposed to play a critical role in the development of human cancers [2, 3]. For instance, aberrant methylation patterns have been attributed to the development of human tumors [4]. Although the mechanism remains incompletely understood, DNA hypermethylation patterns in cancers can be enhanced in silencing tumor suppressor genes, therefore promoting tumor progression [5–7]. The normally unmethylated CpG islands in the promoter regions of some tumor suppressors were found frequently methylated in tumors, which are also correlated with the inactivation of these genes in human cancers [7]. Thus, enhancing promoter methylation such as activating DNA methyltransferase 1 (DNMT1) has been implicated in the pathogenesis and progression of human cancers [8–10]. Furthermore, studying the functions of cancer-related genes, which are transcriptional inactivated by promoter hypermethylation, would boost our understanding of distinctive mechanisms during carcinogenesis in different types of human cancers.

Despite advances in the management of lung cancer over the last several decades, including surgery, chemotherapy, and radiotherapy, it is still one of the cancers with the highest mortality rate and ranks first in cancer-related deaths around the world [11]. Aberrant methylation patterns have been documented in lung tumors associated with potential clinical relevance. For example, the inhibition of the *p16INK4* tumor suppressor via abnormal methylation in its promoter region has been suggested as an early sign during the initiation of NSCLC through the uncontrolled expansion of pre-malignant cells [12–14]. Moreover, as other forms of human cancer, the sequence context of DNA hypermethylation in lung cancer has been examined by high-throughput approaches to find prevalent hypermethylated CpG islands. Many aberrant methylated genes have been identified as candidate molecular markers in lung cancer. Meanwhile, the expression levels of DNA methyltransferases including DNMT1 is frequently elevated in lung cancers, which is significantly associated with the hypermethylation of the p16 promoter [15]. The mechanisms for such elevation are still unclear, but overexpression and activation of DNMT1 or other forms of DNMTs may result in promoter hypermethylation of multiple tumor suppressors, thus ultimately leading to poor prognosis and lung tumorigenesis [16].

DBCCR1 (deleted in bladder cancer chromosome region 1) is a gene whose expression is often reduced in human bladder tumor [17]. Originally proposed as a tumor suppressor gene with loss of heterozygosity occurring in cancer, decreased DBCCR1expression was later attributed to a result of gene silencing through hypermethylation [17–19]. There is, however, lack of information related to DBCCR1 alterations in lung cancers to our knowledge. It has been reported that hypermethylation targeted DBCCR1 occurs in oral squamous cell carcinomas [19], hepatocellular carcinoma [20], and gliomas [21]. DBCCR1 thus likely plays a general role in cancer biology with tumor suppression activity in distinctive cancer types. To study the potential implications of DBCCR1 in lung cancer, we examine the expression of DBCCR1 in patient tissues and cell lines of human lung cancer. The aims of the study also included the further examination of DBCCR1 function in lung cancer cells by genetic manipulation *in vitro*. The study offers an opportunity to study the properties of DBCCR1 in human lung cancers, which may provide a novel molecular target in human patients.

## RESULTS

### Low DBCCR1 expression correlates with tumor progression in lung cancer patients

**Figure 1:**
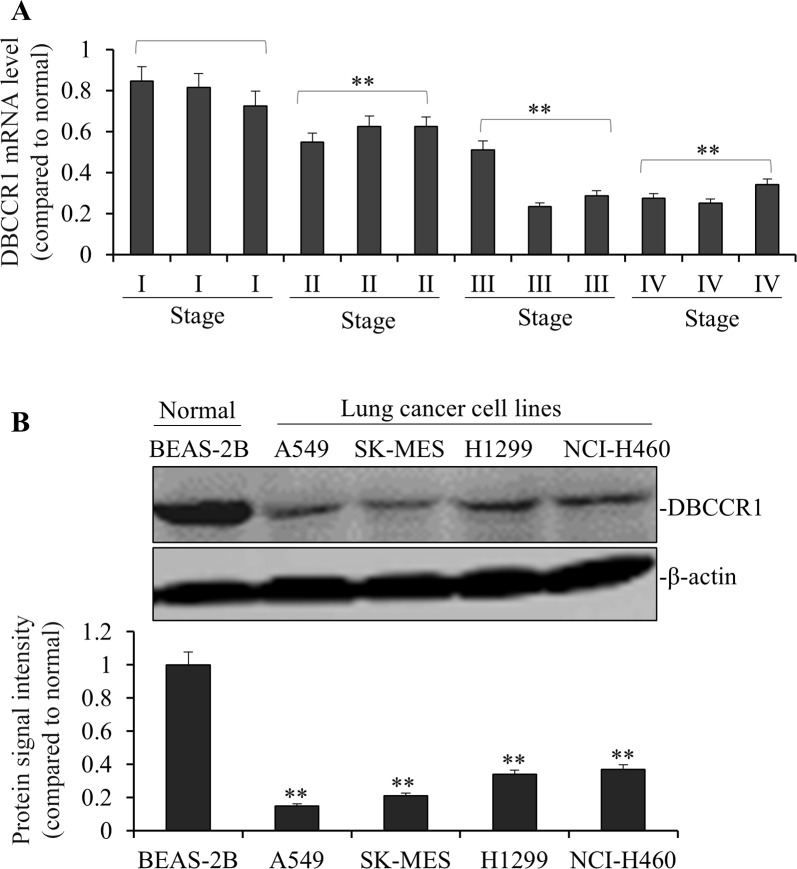
DBCCR1 expression was low in both patient tissue and lung cancer cell lines **(A)** The mRNA levels of *DBCCR1* were low in 12 representative lung cancer patient tissues compared with adjacent non-tumor tissues by PCR. Especially the mRNA levels of *DBCCR1* decreased followed the increase of cancer stages (I, II, III and IV) (*p<0.01*). **(B)** The DBCCR1 protein levels in 4 lung cancer cell lines were normalized to the β-actin protein level and plotted. The data were mean ± SD of three independent experiments. Quantitation by densitometry was shown on below (**P<0.01, compared with normal Human bronchial epithelium cell line-BEAS-2B).

We used quantitative PCR to compare the expression levels of DBCCR1 in lung tumor to the adjacent normal tissues. As such, DBCCR1 expressions were evaluated based on the relative abundance of mRNA levels (tumor to non-tumors). For instance, the subject with a high DBCCR1 level was defined as dramatically higher DBCCR1 expression in tumor tissue than in its corresponding non-tumor tissue. The chi-square test was then applied to analyze the correlation between DBCCR1 expression and clinicopathological features (Table 1). After the patients were divided into two groups based on DBCCR1 levels (arbitrarily determined, 45 low vs 61 high as shown in Table 1), significantly more patients were found containing the low expression of DBCCR1, compared to high DBCCR1 expression, in more advanced stages (III and IV) of tumors. Similarly, when DBCCR1 levels in 12 representative lung cancer patients were specifically analyzed (Figure 1A), low DBCCR1 expressions were correlated with more advanced stages of tumor, with significantly decreased DBCCR1 in late stages of cancer (II, III and IV, *p<0.01* as compared to I, respectively). Comparing to patients with high DBCCR1, low DBCCR1 expression was also correlated with more proliferation marker Ki-67 (*p = 0.000*), tumor metastasis (*p = 0.001*) and tumor invasion (*p = 0.000*). No obvious difference in tumor size was observed between the patients of both groups. To further examine the DBCCR1 expression in lung cancer cells, we compared the level of DBCCR1 protein in normal human lung epithelial cell line BEAS-2B with several human lung cancer cell lines (Figure 1B). The similar association was found in all cancer cells in which DBCCR1 levels were significantly lower than BEAS-2B cells.

**Table 1:** DBCCR1 expression and clinicopathologic characteristics on 102 lung cancer specimens

Characteristics	Total	DBCCR1 expression		P value
		Low	High	
Age (years)				0.567
<60	46	20	23	
>60	56	25	31	
Gender				0.541
Female	60	28	32	
Male	52	26	26	
Tumor grade				0.001*
I	21	5	16	
II	27	10	17	
III	30	17	13	
IV	24	18	6	
Tumor size (cm)				0.341
<5	37	16	21	
>5	65	40	25	
Tumor metastasis				0.001*
Negative	53	9	44	
Positive	49	38	11	
Tumor invasion				0.000*
Negative	27	9	18	
Positive	75	69	6	
Ki-67 expression				0.000*
Low expression	57	12	45	
High expression	44	37	7	
DBCCR1 expression				0.000*
Low expression	41	41	0	
High expression	61	0	61	

Statistical analyses were performed by the SPSS test. * *P*<0.05 was considered significant.

**Figure 2:**
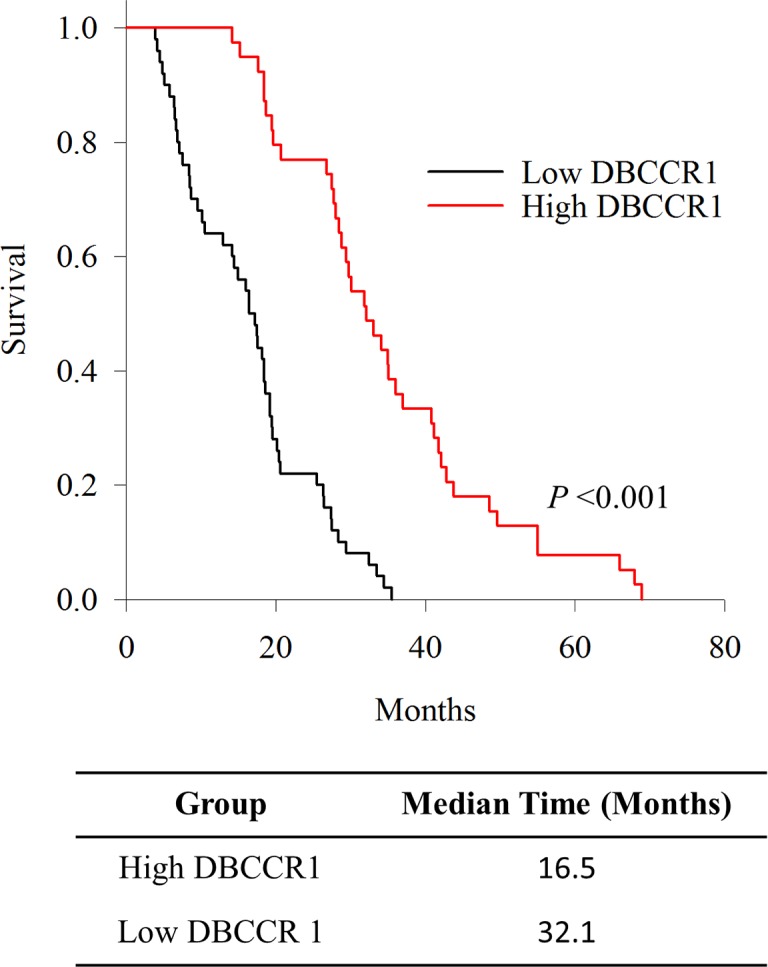
Kaplan-Meier survival curves of DBCCR1 expression status Patients were divided into high and low DBCCR1 expressers according to the basis score of DBCCR1. Patients with high expression group of DBCCR1 had longer overall survival.

Strikingly, the patient group with high DBCCR1 expression had longer overall survival, based on Kaplan-Meier fractions analyzed by log-rank tests (Figure 2). Moreover, Multivariate Cox proportional survival analysis showed that Ki-67 (P = 0.000) and DBCCR1 expressions (P = 0.000), tumor grade (P = 0.001), tumor metastasis (P = 0.001) and tumor invasion (P = 0.000) were independent prognostic biomarkers of overall survival among the patients (Table 2).

**Table 2:** Contribution of various potential prognostic factors to survival by Cox regression analysis in 102 lung cancer specimens

Characteristics	Hazard ratio	95% CI	P value
Age (years)	0.821	0.745–2.078	0.567
Gender	1.154	0.888–1.765	0.541
Tumor grade	1.428	0.865–2.422	0.001*
Tumor size (cm)	0.57	0.347–1.475	0.341
Tumor metastasis	1.081	0.834–1.274	0.001*
Tumor invasion	0.36	0.138–0.541	0.000*
Ki-67 expression	0.233	0.097–0.545	0.000*
DBCCR1 expression	0.67	0.274–1.144	0.000*

Statistical analyses were performed by the Cox regression analysis. * *P*< 0.05 was considered significant

### DBCCR1 repression involves cell growth of lung cancer cells *in vitro*

**Figure 3:**
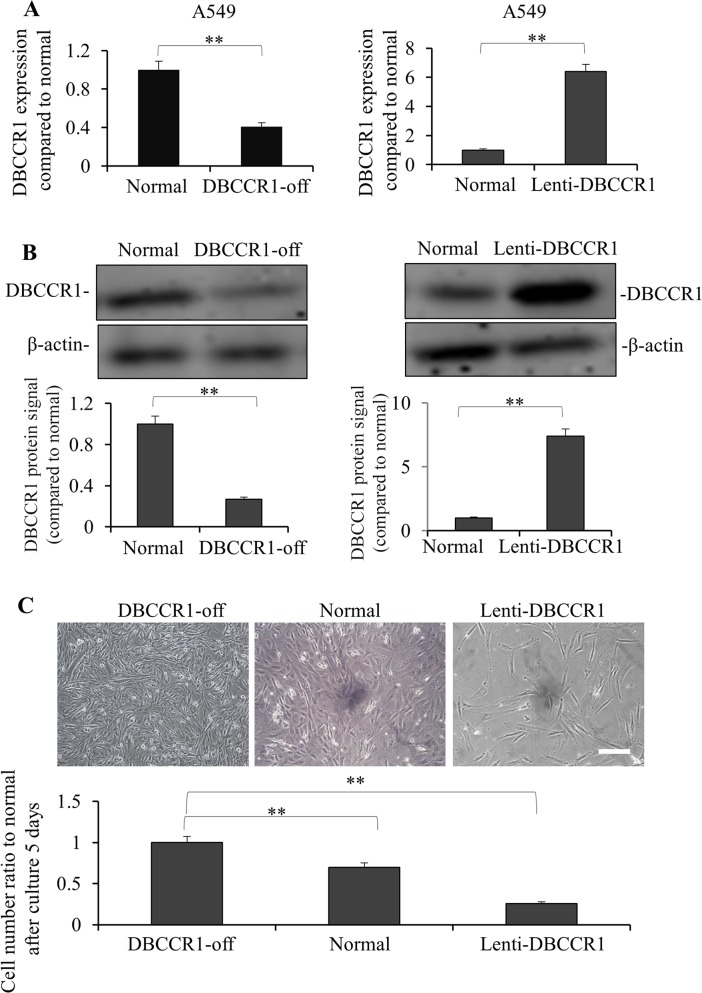
Knockdown and over-expressed of DBCCR1 effected the growth in A549 cell line A549 cells were transfected with DBCCR1-shRNA or control-shRNA for 48 h for knockdown of DBCCR1. A549 cells were infected with Lenti-virus with DBCCR1 or Lenti-virus control for 48 h for over-expression of DBCCR1. DBCCR1 expression was detected by PCR **(A)** and Western blot **(B)**. Quantitation by densitometry was shown on below (**P<0.01, compared with normal cell line). **(C)** The cell growth statuses were observed of DBCCR1-off, over-expressed and normal A549 cells after culture for 5 days by microscopy. The original cell number were the same of 1×10^5^ in 6-well plate. The down-expressed DBCCR1 promoted the tumor cell growth and over-expressed DBCCR1 suppressed the growth. -off, over-expressed and normal A549 cells DBCCR1-off, over-expressed and normal A549 cells. Scale bar 0.5 μm. Quantitation by counting the cell number was shown on below (**P<0.01, compared with normal cell line).

Given the significant correlation between DBCCR1 expression and tumor progression in lung cancer patients, we studied the consequence of human cancer cells *in vitro* when *DBCCR1* gene is altered. In A549 cells, a type of human alveolar basal epithelial adenocarcinoma cells, we decreased or increased DBCCR1expression by lentiviral-mediated shRNA knockdown or constitutively expression, respectively (Figure 3). As Figure 3A (mRNA) and 3B (protein) shown, stable cell lines with either DBCCR1silencing (DBCCR1-off) or DBCCR1 ectopic expression (Lenti-DBCCR1) were successfully established as expected. Interestingly, down regulation of DBCCR1 enhanced, whereas ectopic DBCCR1 expression inhibited the cell numbers of A549 cells as compared to their parental normal cells (Figure 3C). As a background check for the respective controls, we examined the mRNA levels of DBCCR1 in normal (untreated A549 cells), scrambled shRNA transduced (control of DBCCR1-off), and empty vector transduced (control of Lenti-DBCCR1) cells. No significant changes of DBCCR1 level was seen between the groups (Supplementary Figure 1). We thus performed the following analysis in directly comparing normal, DBCCR1-off, and Lenti-DBCCR1 cells.

### DBCCR1 repression promotes cell migration and invasion of lung cancer cells *in vitro*

**Figure 4:**
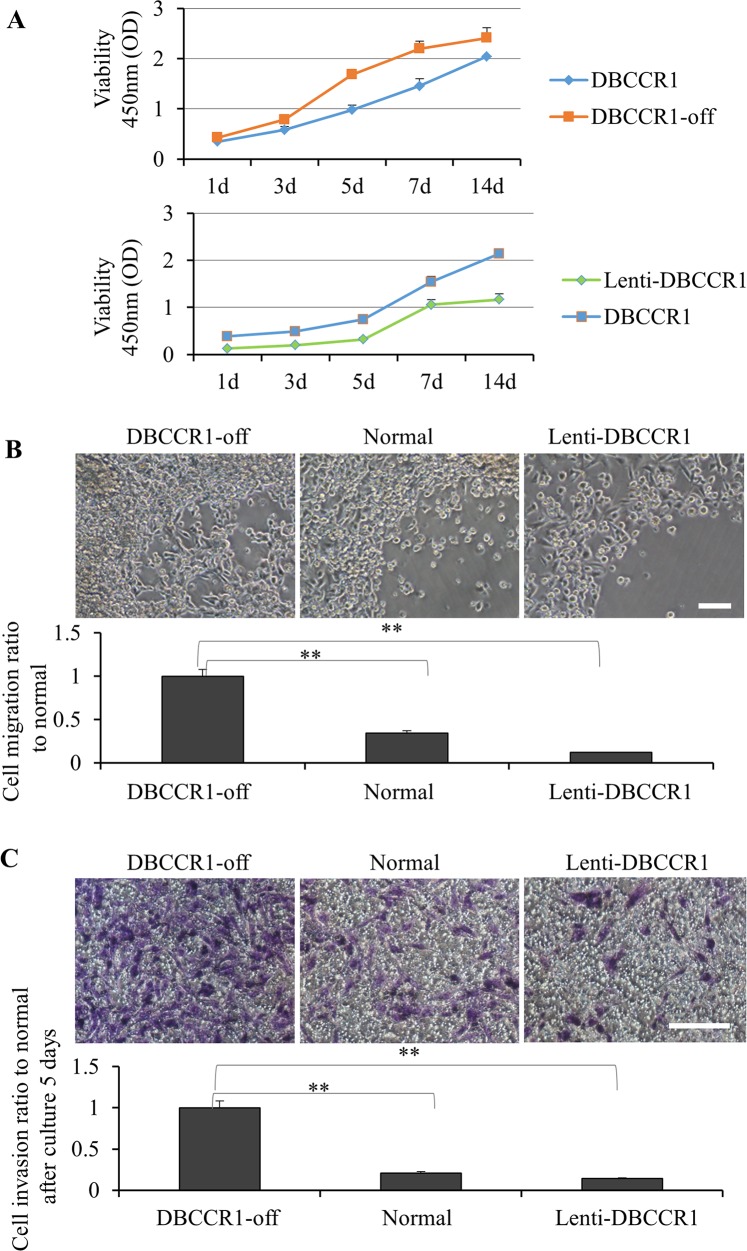
Change the expression of DBCCR1 effected the proliferation, migration and invasion in A549 cell line **(A)** CCK-8 assay was used to detect cell viability of A549 cells treated with DBCCR1-shRNA (compared with control shRNA) and Lenti-virus with DBCCR1. A549 cells were placed on 96-well plates (5×10^3^ cells/well) and incubated with fresh medium. Growth curves were detected. Points and range lines at different day (1, 3, 5, 7 and 14 days) represent mean and SD of at least three independent experiments in triplicate. OD value was measured at 450 nm and data demonstrated a significant growth induction by knockdown of DBCCR1 (*p*<0.01). **(B)** Migration kit assay with DBCCR1-shRNA (compared with control shRNA) and Lenti-virus with DBCCR1 was tested. Migration of the cells to the blank area was visualized at 72 h with an inverted Leica phase-contrast microscope (9200 magnification). Quantitation was shown on below (**P<0.01, compared with normal cell line). Data demonstrated a significant migration capacity induction by knockdown of DBCCR1. **(C)** The relation of DBCCR1 expression and invasion capacity was tested at 72 h after culturing cells by transwell assays. DBCCR1-shRNA cells showed lower penetration rate through the membrane compared with control-shRNA and mock cells. Scale bar 0.5 μm. Quantitation was shown on below (**P<0.01, compared with normal cell line).

We then compared proliferation curves of these cells by cell viability kits (Figure 4A). In A549 cells, silencing of DBCCR1 resulted in greater growth comparing to the scrambled-off cells. In contrast, increasing DBCCR1 in A549 cells reduced the cell proliferation. The oncogenic potential of these cells were also examined *in vitro* by migration and invasion assays. Consistent with a hypothesis that DBCCR1 suppression may regulate tumor progression, DBCCR1-off cells had a stronger response in the ability of cell migration (Figure 4B) and transwell matrigel invasion stimulated by serum (Figure 4C). Reversely, restoring DBCCR1 expression blocked the both reactions in Lenti-DBCCR1 cells, comparing to normal cells (Figure 4B–4C).

### Reciprocal co-regulation of DBCCR1 and DNMT1 in lung cancer

**Figure 5:**
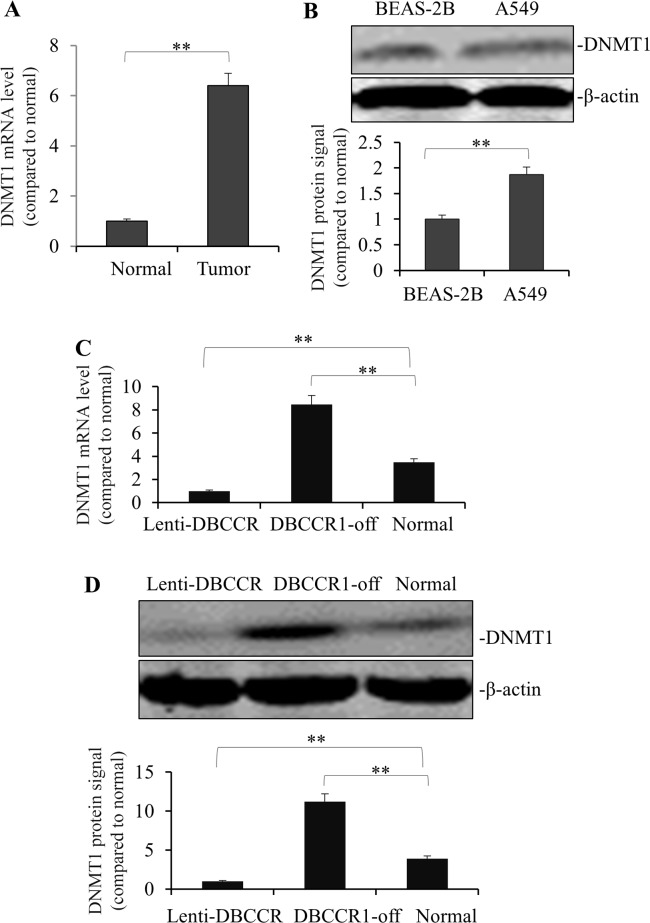
Change of DBCCR1 expression in A549 cells results in reduced counter-trend change of methylation **(A)** The mRNA levels of *DNMT1* were high-rich in 12 representative lung cancer patient tissues compared with adjacent non-tumor tissues by PCR (*p<0.01*). **(B)** The protein levels of DNMT1 were high-rich in A549 compared BEAS-2B by WB. Quantitation by densitometry was shown on below (**P<0.01, compared with non-tumor tissues). A549 cells were transfected with DBCCR1-shRNA or control-shRNA for 48 h for knockdown of DBCCR1. A549 cells were infected with Lenti-virus with DBCCR1 or Lenti-virus control for 48 h for over-expression of DBCCR1. DNMT1 expression was detected by PCR **(C)** and Western blot **(D)**. Quantitation by densitometry was shown on below (**P<0.01, compared with normal cell line).

The above results prompted us to speculate that effects of DBCCR1 in lung cancer cells could be related to a change of DNA methylation. To test this, DNMT1 expression was measured in the tumor tissues. As Figure 5A shows, mRNA of DNMT1 is highly expressed in tumors compared to normal tissues. This finding was also consistent with an observation of higher DNMT1 protein level in A549 cells than normal lung cell line BEAS-2B (Figure 5B). Finally, we compared the expression levels of DNMT1 (mRNA in Figure 5C and protein in Figure 5D) in genetically-manipulated A549 cells. Intriguingly, DNMT1 expression was dramatically blocked in Lenti-DBCCR1 cells, whereas in DBCCR1-off cells there was a remarkable induction of DNMT1 level both in RNA and protein levels (Figure 5C–5D).

## DISCUSSION

[2, 8, 9, 20] In human lung cancer, elevated mRNA expression of DNMT1 may be an independent and important prognostic factor and constitute a useful biomarker for early detection, monitoring, and treatment of cancer patients [4, 22]. Furthermore, increasing activity of DNA methylation driven by elevated DNMT1 expression in lung cancer could contribute to pathogenesis and progression of tumors through the CpG island hypermethylation of cancer suppressors. Nevertheless, there is a lack of information in lung cancers regarding the mechanism of DNMT1 upregulation. On the other hand, the methylation status of human tumors has been characterized extensively with distinct methylation profiles from specific tumor types [23, 24]. Interestingly, prior data suggested that cancer related microRNAs can promote aberrant DNA methylation in tumors via targeting DNMT1 [25]. It thus has been speculated that some tumor suppressors repressed by hypermethylation may block DNA methyltransferase itself. In this study we tested this hypothesis by showing DBCCR1, a potential methylation target in bladder cancers, is decreased in human lung cancers and associated with an elevation of DNMT1. When DBCCR1 was genetically manipulated in a human lung cancer cell line, we found that DNMT1 expression was reciprocally modulated, implicating a more complex interaction of cancer-associated gene repressions with transcriptional up-regulation of DNA methylation. These findings therefore have a potential to explain the coincidence between an increase of DNMT1 and a decrease of DBCCR1 expression we showed in both patient tissues and cancer cell lines. By demonstrating DBCCR1-dependent DNMT1 gene regulation in human cancer cell lines, we provide evidence to suggest DBCCR1 repression may serve as a molecular switch that stimulates up-regulation of DNMT1 in lung cancer. Future studies focusing on signaling such as DBCCR-DNMT1 axis could further reveal mechanisms of tumor pathogenesis via the complementary interplays between tumor suppressors and aberrant hypermethylation.

The exact function of DBCCR1 protein has yet been completely defined. Highly expressed in various brain regions of adult mice, DBCCR1 expression is markedly induced by neural activity. Gene ablation of DBCCR1 in mice results in impaired neurogenesis and abnormalities in behaviors that could be due to defective neuronal differentiation in hippocampal circuitry [26]. In cancers, early findings documented the homozygous deletions at the 9q32-33 region (containing DBCCR1 loci) and absence of *DBCCR1* mRNA expression in bladder tumors [17, 18]. In addition, the ectopic expression of DBCCR1 in murine cells and human bladder cancer cell lines appeared to promote cell proliferation and result in an increase in the G1 phase without perturbing cell apoptosis [27]. Similarly, transient transfection of a GFP-DBCCR1 construct in bladder cancer cell lines induces cell death which is not the classic apoptotic type [28]. These observations imply DBCCR1 could be a candidate tumor suppressor gene in bladder cancer. Consistent with this notion, our current data showed that growth ofA549 lung cancer cells was dependent on DBCCR1 repression *in vitro*. In addition, we surveyed the ability of cancer cells in migration and invasion which appeared to require the suppression of DBCCR1. The data support the oncogenic potential of lung cancer cells might be caused by the down-regulation of DBCCR1. To perform the *in vitro* assays, we generated stable DBCCR1-expressing A549 cells using a lentiviral-mediated approach. This observation is different from a previous study in bladder cancer cell lines [28] where stable DBCCR1 expression was unsuccessful. The discrepancy is likely due to variations of the inhibition effects of DBCCR1 overexpression on *in vitro* growth of different tumor types. Collectively, these findings support a role for DBCCR1 silencing in carcinogenesis of human lung cancer, possibly through enhancing cell proliferation and cell migration. The *in vivo* impacts of these processes need to be confirmed and characterized in the future, such as in an animal xenograft model of lung cancer. The implications of DBCCR1-dependent regulation in lung cancer also deserve further in-depth clinical investigation. For example, whether or not the promoter hypermethylation is associated with the reduced DBCCR1 expression in lung cancers is still an open question. As we showed in the current study the levels of DBCCR1 expression are highly related to the outcomes of lung cancer patients, including the stage of cancer and survival of subjects, a larger cohort of patients needs to be examined to verify this intriguing finding.

How DBCCR1 may inhibit DNMT1 expression is an important question. No conceivable pathways were identified at this point. Previously, DBCCR1 has been only suggested as a potential target of DNMT1-dependent methylation during development [29]. In a most recent report [30], the gene locus of DBCCR1 has been associated with DNMT1 activity in cancer. In this study, Qi D *et al*. found a correlation between low DBCCR1 level and high expression of DNMT1 in bladder cancer tissues. Their data support a direct inhibition of DBCCR1 through the DNMT1-mediated methylation of DBCCR1 promoter, which can be prevented by a long non-coding RNA derived from the DBCCR1 locus itself [30]. Complementary to these findings, the results of our study implied that DBCCR1 suppression and DNMT1 activation could be synergistically induced during tumor progression via positive feed-forward mechanisms. Following this rationale, it is of critical importance to investigate if DNMT1 is necessary for the stimulated cancer cell growth and invasion in DBCCR1-deficient cells. Given the prior observations that upregulation of DNMT1-mediated gene silencing can promote tumor cell migration and metastasis in several types of cancers [22, 31], we speculate that DNMT1 induction, at the least partially, is responsible for the phenotype of cancer cells with reduced DBCCR1 expression. The results may illustrate the intricate functions of a potentially common pathway leading to tumor progression in multiple cancers.

Our study shows that low expressions of DBCCR1 correlate with severe tumor progression and poor outcomes of lung cancer patients. *In vitro* manipulations of DBCCR1 expression alter the cell proliferation, migration, and invasion of lung cancer cell line A549. Finally, DBCCR1 may inhibit DNMT1 induction in lung cancers. The results thus suggest DBCCR1 gene that has reduced expression in lung tumors has a potential tumor suppressor function.

## MATERIALS AND METHODS

### Lung cancer specimen collection

Patients with lung adenocarcinoma recruited in the study were aged 34-78 years old. Tumor stages were determined following the American Joint Committee on Cancer TNM (tumor, nodes, metastasized) system. All patients have signed written informed consent forms, and the study protocol was approved by ethics committees of Jiangsu Cancer Hospital Affiliated to Nanjing Medical University following the guidelines of Declaration of Helsinki and Good Clinical Practice. For the correlation study, tumor and adjacent normal tissues of patients were collected during the surgery and snap-frozen for storage at -70°C. Pathologic staging of lung cancer patients was determined, and histological evaluation of tumor tissues using Ki-67 proliferation marker was also included. The survival curves of the time to the decease of cancer patients analyzed, and based on the expression levels of DBCCR1, were calculated by use of the Kaplan-Meier method.

### Cell culture and reagents

Normal (BEAS-2B) human lung cells or human lung cancer cell lines (including A549, SK-MES, H1299, and NCI-H460) were obtained from ATCC, and cultured in the complete DMEM growth media containing 10% fetal bovine serum at 37°C with 5% CO_2_.

### RNA extraction and quantitative PCR

We assessed the mRNA levels of gene expression by quantitative real-time PCR. Total RNA from patient tissues or cultured cells were isolated by TRIzol (Invitrogen, Carlsbad, CA, USA), and reverse transcribed to complementary cDNAs using Superscript II (Biorad, Hercules, CA, USA) based on manufacturer's instructions. SYBR Green PCR Master Mix assay (Applied Biosystems, Waltham, MA, USA) was used for detection. Standard curves were generated from a series of dilutions of control cDNAs to validate linear ranges and melting curves for each gene primer set. Specific primers used included *DBCCR1*, forward 5′-GGGAGGTAGAGGGAGTAGTGAT-3′, reverse 5′-AAAATACCTAACTCCTAACAACCTACC-3′; *DNMT1*, forward 5′-AGGCGGCTCAAAGATTTGGAA-3′, reverse 5′-GCAGAAATTCGTGCAAGAGATTC-3′; *GAPDH*, forward 5′-AATGGACAACTGGTCGTGGAC-3′, reverse: 5′-CCCTCCAGGGGATCTGTTTG-3′. Triplicated PCR reactions were performed for each sample. Minus reverse transcriptase samples were used as negative controls, and *GAPDH* was used as a housekeeping gene for normalization.

### Western blot

Western blotting was performed in tissue samples or cultured cells as indicated. After the cells were lysed in buffer containing 1% NP40, 50 mM Tris, 5 mM EDTA, 1% sodium deoxycholate, 1% SDS, 1% Triton X-100, 10 mg/ml aprotinin, 1mM PMSF, 1 mg/ml leupeptin, and pH=7.5, supernatants were collected after spin and protein was measured by Bradford assay (Thermo, Waltham, MA, USA). Forty micrograms total proteins were resolved on SDS-PAGE. Following an electric transfer onto PVDF membranes, the blots with proteins were then blocked by 5% bovine serum albumin and incubated with appropriate primary antibodies at 4°C overnight. The membranes were then incubated by HRP conjugated secondary antibody, and signals were visualized by an enhanced ECL-based imaging system. Antibodies used in the study include DBCCR1 (1:1000, Abcam, Cambridge, MA, USA), DNMT1 (1:1000, Cell signaling, Danvers, MA, USA) or β-actin (1:5000, Sigma, St. Louis, MO, USA). The graphs shown are representative images from three independent experiments. Quantitation by densitometry was performed in ImageJ and normalized by β-actin. The results were compared with normal human bronchial epithelium cell line-BEAS-2B.

### Knockdown and overexpression of DBCCR1

To knock down DBCCR1 expression in A549 cells, a lentiviral-mediated shRNA expression method was carried out. The lentivirus containing DBCCR1-specific shRNA (CCTGCTCCCATTAAGACGCTCTAAAGGGAAAAAAA) was generated by transfection of pLKO.1-based lentiviral shRNA expression plasmids (scrambled control or DBCCR1-specific) along with helper plasmids (the packaging plasmid psPAX2 and envelope plasmid pMD2) in 293t cells (using Lipofectemin2000, Invitrogen, USA). A549 cells were then incubated in 48-hour conditioned media of 293 containing the virus (polybrene 10 μg/ml) for another 2 days before the following assays were performed. To overexpress DBCCR1 by similar approach, A549 cells were infected with lentivirus with human DBCCR1 cDNA (NM_014618, based on CMV promoter) or empty vector controls for 48 hours before the following assays were performed. In both experiments, cells were selected under G418 treatment.

### Cell growth and proliferation assay

Cell growth was assessed in 6-well plates (starting at 1×10^5^/well), and cells were counted after 5-day culture by microscopy. Representative photos of cells were taken under a light microscope. Viability of cells was further assessed by CCK-8 assays over a period of time. The same cell numbers (5×10^3^ cells/well in 96 well-plates) were cultured and cell viability was determined at different day (1, 3, 5, 7 and 14 days) by incubation with CCK-8 reagent (100 μl per well). The absorbance at 450 nm was then measured by a spectrometer and the data was normalized as relative numbers.

### Cell migration and invasion assays

The ability of cell migration was assessed by a wound closure assay. In brief, a wound was generated as the cell boundary at time zero by scraping confluent cells with a 200 μl pipette tip. 24 hours later, the migrating cells into the open areas were analyzed in an inverted Leica phase-contrast microscope. The results of migration were normalized to the control cells. The ability of cell invasion was examined in a Boyden chamber assay. 1 × 10^5^ cells in 300 μl serum-free media were seeded into each well on 8 μm-pore polycarbonate membrane transwells (Costar, USA) pre-coated with Matrigel (BD Biosciences, Franklin Lakes, NJ, USA). The transwells were cultured for 24 hours with the lower chambers in 700 μl DMEM supplemented with 10% FBS. After wiping off the cells on the top surface of the membrane with a cotton swab, we counted the cells that migrated to the bottom surface of the membrane. Cells were fixed in methanol and stained by crystal violet. Cells in 6 random areas were photographed and counted. Representative photos of cells were taken and the results represent the average number of three repeated experiments normalized as relative numbers.

### Statistical analysis

All data were expressed as mean ± standard deviation (SD) from the results of at least three independent experiments. One-way ANOVA was used to analyze the significance of differences between cancer cell and normal cell lines. For the correlation study, results were analyzed by chi-square test. Patient survival was analyzed by Kaplan-Meier fractions, and compared by Logrank tests. Student t-tests were used to compare knockdown or overexpression of DBCRR1 in A549 cells with the appropriate controls, respectively. Cell growth curves were compared with two-way ANOVA. A *P* value < 0.05 was considered as statistically significant difference.

## SUPPLEMENTARY FIGURE


