# Supplementary material for: Identification of DBCCR1 as a suppressor in the development of lung cancer that is associated with increased DNA methyltransferase 1
Source: Oncotarget. 2017 Mar 2;8(20):32821–32. doi: 10.18632/oncotarget.15826 (PMC5464830; doi:10.18632/oncotarget.15826)
Supplement: Supplementary file 1 [file oncotarget-08-32821-s001.pdf]

Identification of DBCCR1 as A Suppressor in the Development of Lung Cancer that Is Associated with Increased DNA Methyltransferase 1

SUPPLEMENTARY FIGURE

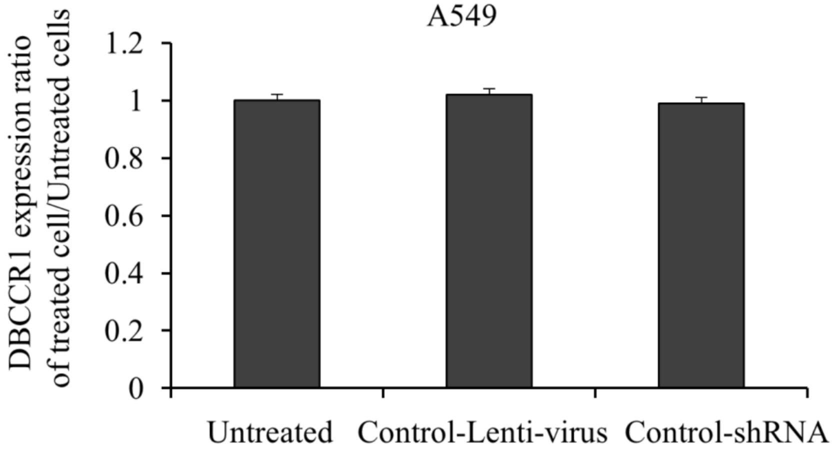

Supplementary Figure 1: The difference between untreated A549 and control of knockdown/overexpression DBCCR1. A549 cells were transfected with shRNA vector or for 48 h as control of knockdown of DBCCR1. A549 cells were infected with Lenti-virus without DBCCR1 for 48 h as control of over-expression of DBCCR1. DBCCR1 expression was detected by PCR. The data showed no obviously difference of three cell lines.
